# Antibiotics with antibiofilm activity – rifampicin and beyond

**DOI:** 10.3389/fmicb.2024.1435720

**Published:** 2024-08-29

**Authors:** Luís Ferreira, Ema Pos, Daniela Rodrigues Nogueira, Filipa Pinto Ferreira, Ricardo Sousa, Miguel Araújo Abreu

**Affiliations:** ^1^Department of Infectious Diseases, Centro Hospitalar e Universitário de Santo António, Porto, Portugal; ^2^Department of Orthopaedic Surgery, Centro Hospitalar e Universitário de Santo António, Porto, Portugal; ^3^Grupo de Infeção Osteoarticular do Porto, Porto, Portugal

**Keywords:** prosthetic joint infection, biofilm, antibiofilm activity, antibiotic therapy, biofilm active antibiotics, drug resistance

## Abstract

The management of prosthetic joint infections is a complex and multilayered process that is additionally complicated by the formation of bacterial biofilm. Foreign material provides the ideal grounds for the development of an intricate matrix that hinders treatment and creates a difficult environment for antibiotics to act. Surgical intervention is often warranted but requires appropriate adjunctive therapy. Despite available guidelines, several aspects of antibiotic therapy with antibiofilm activity lack clear definition. Given the escalating challenges posed by antimicrobial resistance, extended treatment durations, and tolerance issues, it is essential to ensure that antimicrobials with antibiofilm activity are both potent and diverse. Evidence of biofilm-active drugs is highlighted, and alternatives to classical regimens are further discussed.

## 1 Introduction

**Figure 1:**
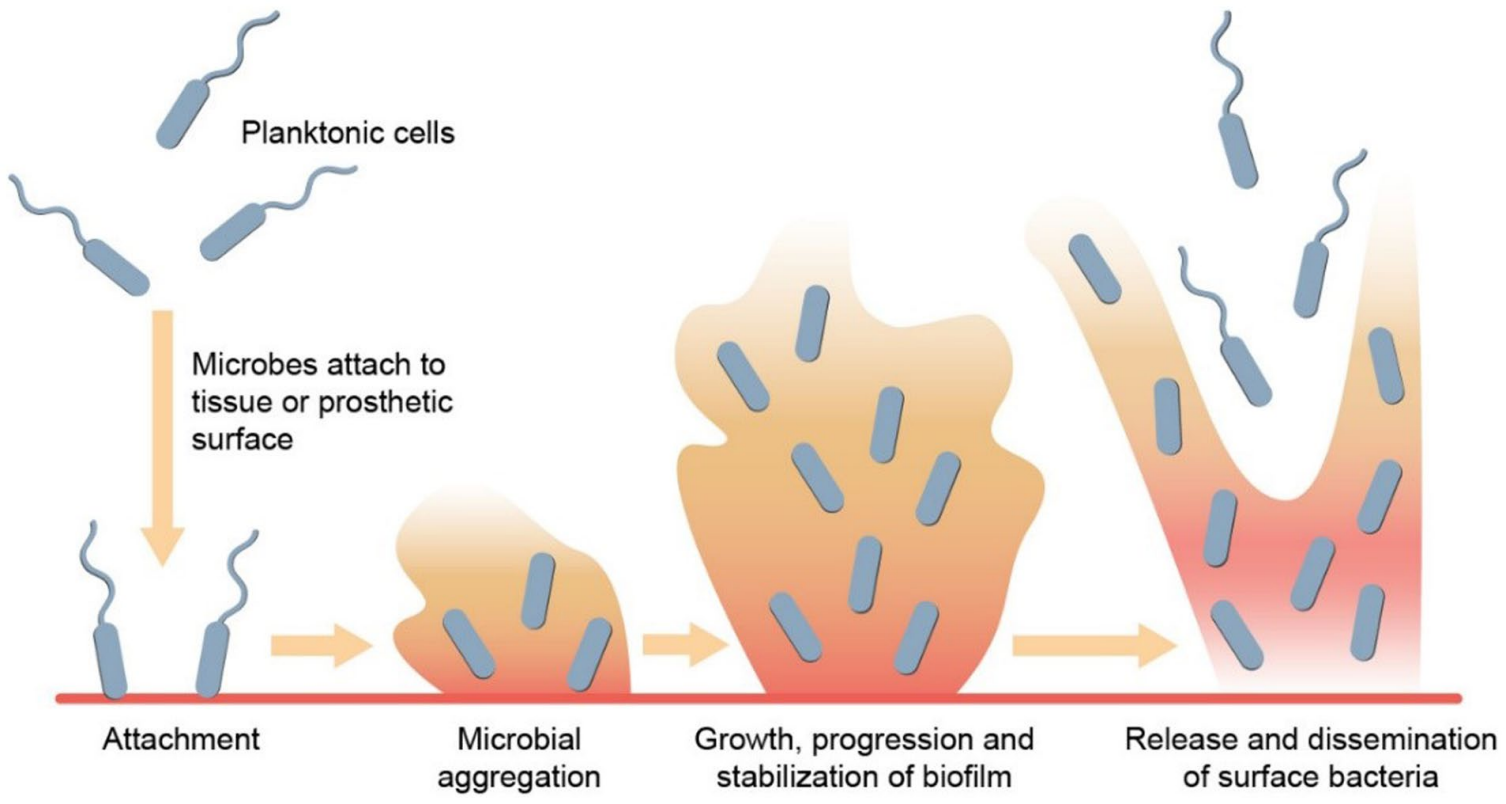
Biofilm formation. Biofilm development begins with planktonic cells initially attaching reversibly to a surface. These cells then attach irreversibly, resulting in the establishment of a bacterial colony on the surface. Through quorum sensing and various signaling mechanisms, the biofilm matures, and stabilizes. Subsequently, microbes within the biofilm disperse by releasing surface bacteria from the top layer, enabling them to colonize new surfaces.

Bacterial involvement has been described in three different states – planktonic, biofilm and intracellular (McConoughey et al., 2014). Biofilm formation is an important mechanism of bacterial pathogenesis, which is characterized by the clustering of (usually monomicrobial groups of) microorganisms on a biological or synthetic surface that form a network of extracellular polymeric substances. As opposed to extracellular bacteria in a planktonic state, which are freely dispersed in the environment, thus vulnerable to aggression, those in biofilm find strength in numbers. Through a mechanism known as quorum sensing, colonies of bacteria transition from planktonic state to biofilm formation once the colony is large enough. This ultimately improves their collective chance of survival through various mechanisms that culminate in antibiotic tolerance, including physical and chemical barrier formation, metabolic adaptation, and genetic material exchange (Figure 1).

Foreign materials such as orthopaedic hardware provide a suitable environment for bacteria to organize and develop a biofilm. Periprosthetic joint infections (PJIs) are a well-recognized and serious complication of prosthetic joint implantation. The importance of biofilm formation in PJIs is clinically illustrated by the decreasing success of interventions involving prosthesis retention as time since infection onset increases and it is no coincidence that some of the most common pathogens, such as Staphylococci and Streptococci, but also the problematic and often multidrug-resistant *Pseudomonas aeruginosa* are well known for their biofilm formation capabilities (McConoughey et al., 2014).

In many centres, the incidence of PJIs varies from 0.5 to 2 %, depending on the afflicted site (Namba et al., 2013). PJIs can stem from various sources, such as surgical contamination, hematogenous dissemination or direct spread from contiguous infection (Ahmed et al., 2019). The most common microorganisms involved in PJIs are gram-positive pathogens, mainly Staphylococci when considering hip and knee interventions and *Cutibacterium acnes* and Streptococci following shoulder arthroplasties. Gram-negative pathogens are also relatively frequent, particularly in the setting of comorbidities or previous antibiotic exposure (Tande and Robin, 2014).

Clinical detection of PJIs can often be difficult considering the indolent course of the chronic infection, relatively low-culture yield of low-virulence bacteria and the ample variety of microorganisms and antibiotic-resistance patterns that can occur. The diagnosis and management of PJIs is, therefore, a complex and multi-faceted process that requires a multidisciplinary approach, encompassing specific medical and surgical strategies, adapted to the clinical scenario and patient involved (Vavasseur and Zeller, 2022). Clinical experience and animal models indicate that intensive debridement is the cornerstone of effectively clearing PJIs and that systemic antibiotic therapy is most effective when started as soon as possible after surgical revision and when combined with local antibiotics (Li et al., 2022). Additionally, accurate identification of the microorganisms involved and their susceptibility to antibiotics is essential for favourable outcome (Zeller et al., 2018).

Treatment of PJIs requires optimal antibiotic coverage, particularly during the initial post-operative phase. Initial therapy usually involves high-dose antibiotics, followed by coupling with drugs possessing antibiofilm properties. The choice of antimicrobial for treating biofilm-associated infections needs to weigh in several factors: tissue and biofilm penetration, possible routes of administration, pharmacokinetic/pharmacodynamic (PK/PD) parameters, tolerability, drug–drug interactions, among others. Rifampicin and fluoroquinolones have been the most used and investigated drugs in this context, becoming a staple in the treatment of PJIs. However, emerging resistance patterns, drug interactions, and adverse reactions profile has highlighted the need for alternative therapies. To date, large clinical and comparative studies with other antibiofilm drugs are lacking, and the role for these is still yet to be defined.

Multiple techniques have been adopted to simulate biofilm formation and assess the antibiofilm properties of antimicrobials. Most studies have relied on *in vitro* biofilm models, which can be both diverse and complex. Nonetheless, *ex vivo* and *in vivo* models have also been put into practice.

Biofilm formation can be assessed by combining various methods, such as staining with conventional, immune histochemical or fluorescent dyes, with direct visualization through light microscopy, scanning electron microscopy or confocal scanning laser microscopy. Other methods include fluorescent *in-situ* hybridization, piezoelectric sensing, and infrared spectroscopy. Bioluminescence assays and nuclear imaging techniques allow for non-invasive *in vivo* assessment but are not specific to biofilm formation (Roy et al., 2018). To evaluate potential antibiofilm effects of specific compounds, studies rely on counting colony forming units before and after the compounds are added to the chosen experimental system.

As for antibiotic susceptibility testing, there is no satisfactory assessment to date. Several concepts have been suggested, such as the minimal biofilm eradication concentration (MBEC), the minimal biofilm inhibitory concentration (MBIC), the biofilm bactericidal concentration or the biofilm prevention concentration, but both definitions and thresholds for interpretation vary significantly. The following article reviews the current knowledge on antibiofilm activity and effectiveness of existing mainstream therapy (fluoroquinolones and rifampin), along with the proposed efficacy of alternative antibiotics that could be considered as therapeutic options. Alternate therapies, such as antimicrobial peptides, bacteriophages, probiotics and plant extracts, have not been considered for review since there is no robust evidence for their use.

## 2 Antibiotics with antibiofilm activity

### 2.1 The paradigm – rifampicin

Rifamycins include rifampicin, rifabutin, rifapentine and rifaximin, though the first stands out as the most employed in the treatment of PJIs. The inhibition of microorganisms’ RNA polymerase confers rifampicin its bactericidal effect (Thill et al., 2022; Eriksson et al., 2023). Its known ability to penetrate biofilm and the possibility for oral administration make rifampicin a powerful ally in single-stage interventions for PJIs, such as debridement, antibiotics, and implant retention (DAIR) strategies. However, despite being active against some gram-negative microorganisms, rifampicin’s role in PJIs is limited to infections by gram-positive bacteria. Lastly, rifampicin is a safe drug. However, drug–drug interactions are frequent, and its use can lead to anaphylaxis, haemolytic anaemia and thrombocytopenia, acute renal failure, rash, hepatic toxicity, and gastrointestinal symptoms, the latter being the most common (Roblot et al., 2007; Nguyen et al., 2015).

Albano et al. investigated *in vitro* activity of rifampicin, rifabutin, and rifapentine against Staphylococci, Streptococci and Enterococci in both planktonic and biofilm states in the setting of PJIs. They found that except for some *Streptococcus mitis* group isolates, antibiofilm activity was significantly higher in staphylococcal infections versus streptococcal and enterococcal PJIs (Albano et al., 2021). These results are supported by a more recent analysis in South Korea, where rifampicin and other rifamycins showed promising antibiofilm effects against *in vitro* samples of patients with PJIs (Lee et al., 2022).

These encouraging *in vitro* results echo in the clinical setting. Significantly lower rates of infection relapse with therapeutic regimens that included rifampicin have been observed in PJIs patients with rifampicin-sensitive pathogens (Lazarinis et al., 2023). Notwithstanding, some groups still question whether rifampicin has real benefit in PJIs.

In one randomized controlled trial (RCT), the addition of rifampicin to standard antibiotic therapy failed to reveal a statistically significant advantage (Karlsen et al., 2020). Renz and colleagues dissected this controversy, who underlined the existence of abundant evidence, starting from the very first scientific observation of rifampicin’s antibiofilm activity in animal models in 1983 (Renz et al., 2021). Additionally, a recent study by Beldman et al. corroborated the benefit of rifampicin in cases of acute PJIs treated with DAIR, particularly in knee infections (Beldman et al., 2021). It has also been demonstrated that treatment failure can be up to four times more likely in infections caused by rifampicin-resistant agents, as opposed to rifampicin-sensitive microorganisms (Lazarinis et al., 2023).

Though this may be a testament to rifampicin’s worth in the treatment of PJIs, it also brings us to the matter of antibiotic resistance.

Rifampicin resistance can develop fast and is intrinsically related to a mutation in the rpoB gene encoding the β-subunit of bacterial DNA-dependent RNA polymerase (Lee et al., 2022). Some pathogens, namely Staphylococci, carry the genes coding for these resistance mechanisms and there appears to be a growing tendency in antibiotic-resistant strains of Staphylococci, particularly in the setting of PJIs. This represents a relevant contributing factor in treatment failure (Lazarinis et al., 2023). A study by Eriksson et al. highlighted that in cases of PJIs due to rifampicin-resistant bacteria, there was a clear increase in the number of surgical interventions required for treatment success, in contrast with rifampicin-sensitive pathogens, thus underlining antibiotic resistance as an important factor contributing to prolonged periods of treatment and further complications requiring multiple interventions (Eriksson et al., 2023).

#### 2.1.1 Practical considerations

Strategies for adequate prescription and prevention of rifampicin resistance include antimicrobial susceptibility testing, careful consideration of the timing for initiation and combination therapy.

As described above, methods to evaluate antimicrobial susceptibility in objective biofilm models are still far from applicable in the clinical setting. Thill et al. developed a study that aimed to compare the usage of minimal inhibitory concentration (MIC), minimum bactericidal concentrations (MBC) and MBEC of rifabutin and rifampicin in cases of PJIs caused by Staphylococci. Their results show advantage in using MBEC values. However, further *in vivo* studies are necessary (Thill et al., 2022). In the meantime, conventional susceptibility testing is highly recommended.

There has been ongoing dispute about the optimal moment to start rifampicin in the context of PJIs. Intuitively, one would want to start antibiofilm therapy as soon as possible to prevent the maturation of the biofilm after thorough debridement. However, an exceedingly early start in rifampicin can lead to antibiotic resistance and adverse outcomes (Sendi and Zimmerli, 2017). The risk of development of rifampicin resistance is higher in the presence of high bacterial load, which is frequently the case shortly after surgery. As such, it is advisable to slightly delay rifampicin introduction, typically 3–5 days after debridement is completed, surgical drains have been removed and there is no observable drainage from the wound. This ensures significant decrease of bacterial load and lower risk of developing resistance.

There is no consensus about the optimal dosage of rifampicin when treating PJIs. Within the realm of staphylococcal PJIs, some authors support using a fixed daily dose, while others favour an adjustment according to the patient’s weight, and others still prefer a twice-daily scheme (Lora-Tamayo et al., 2013). A study by Nguyen et al. defends an overall benefit in prescribing rifampicin as a single daily dose of 600 mg in combination with levofloxacin (Nguyen et al., 2015).

Lastly, due to the risk of resistance, rifampicin should not be prescribed as monotherapy. Rifampicin combination therapy is associated with better clinical outcomes in infections with established biofilms (Forrest and Tamura, 2010).

Further research is essential to better understand the incidence and determining factors of rifampicin resistance, more specifically in PJIs, the ideal dosage and the potential associations with other medications. While there appears to be an advantage in using rifampicin and in slightly delaying its start, further research would aid in shedding light on the ideal timing. Furthermore, research into tailored interventions considering infection site, aetiology, surgical approach, and antimicrobial therapy will surely contribute to an improvement in specialized optimal care.

### 2.2 Other antibiotics

#### 2.2.1 Antibiotics active against gram-positive and gram-negative microorganisms

##### 2.2.1.1 Fluoroquinolones

Fluoroquinolones (FQs) are broad-spectrum antimicrobials which inhibit bacterial DNA synthesis through the inhibition of DNA gyrase and topoisomerase IV, its primary target against gram-positive and gram-negative bacteria, respectively. FQs are most potent against gram-negative bacilli, such as *Enterobacter* spp. and *Pseudomonas aeruginosa*, but their spectrum includes selected gram-positive bacteria, anaerobes, and mycobacteria.

FQs are available in oral and intravenous (IV) formulations and exhibit excellent bioavailability and tissue penetration, particularly within bone and synovial fluid (Thabit et al., 2019). Additionally, bone penetration of levofloxacin does not seem to be affected by level of ischemia (Lozano-Alonso et al., 2016). Despite their good tolerability and safety profile, severe adverse effects have been associated with their use, and should be kept in mind. These include altered mental status, peripheral neuropathy, secondary *pseudotumor cerebri*, tendinopathy and dysglycaemia (Aspinall et al., 2009; Bidell and Lodise, 2016; Sodhi et al., 2017) Observational studies have also suggested that there is an increased risk of aortic aneurysm and dissection, motivating the American Food and Drug Administration to emit a black label warning in 2018 advising against the use of FQs in individuals with risk factors for aneurysms. However, there appears to be a confounding effect (Brown et al., 2023). FQs should be avoided in patients with myasthenia gravis, as muscle weakness might be exacerbated (Krenn et al., 2020). Moreover, the FQs are associated with a greater risk of *C. difficile* infection.

Robust *in vitro* and clinical evidence, along with their high bioavailability, prolonged half-life, and post-antibiotic effect, as well as adequate penetration in bone tissue and collections, make quinolones one of the preferred options to treat PJIs. Subsequently, many guidelines recommend FQs as a first-line option for targeting biofilm by gram-negative microorganisms and for combination therapy with rifampicin for gram-positive pathogens, including Methicillin-Resistant *Staphylococcus aureus* (MRSA) (Osmon et al., 2013; Høiby et al., 2015).

The FQs bactericidal activity has been shown to be independent of cell growth rate and the antibiofilm activity of these antimicrobials has been demonstrated in *in vitro* studies, particularly with *Pseudomonas aeruginosa* and *Escherichia coli* models (Wiestner et al., 1991; Tanaka et al., 1999). This activity has been further observed in human trials, justifying its pivotal role in the management of gram-negative PJIs (Rodríguez-Pardo et al., 2014; Høiby et al., 2015). In retrospective studies analysing PJIs due to gram-negative bacilli, Martínez-Pastor et al. and Rodríguez-Pardo et al. found that regimens including a fluoroquinolone were associated with better outcomes (Martínez Pastor et al., 2009; Rodríguez-Pardo et al., 2014).

The quinolones’ effect on biofilm appears to be potentiated by its combination with rifampicin, as has been demonstrated in both pre-clinical (Muller-Serieys et al., 2009; Holmberg et al., 2012; Meléndez-Carmona et al., 2022) and clinical studies, particularly in the case of early PJIs (Zimmerli et al., 1998; Berdal et al., 2005; Laffer et al., 2006; Barberán et al., 2008; Senneville et al., 2011). However, regular use of FQs may be threatened by a significant increase in resistance (Martínez Pastor et al., 2009; Benito et al., 2016). Thus, empirical treatment with ciprofloxacin as monotherapy should be avoided to minimize selection of resistance in high bacterial load infections, which has been associated with a sharp decrease in successful treatment in the context of DAIR (Rodríguez-Pardo et al., 2014). When combined with rifampicin, levofloxacin and moxifloxacin were shown to be more advantageous when compared to ciprofloxacin, clindamycin and cotrimoxazole, the latter being associated with the highest rate of treatment failure (Beldman et al., 2021).

Doubts remain on whether FQs’ increased efficacy against biofilms is due to increased bactericidal activity (Blaser et al., 1995) or due to fluoroquinolone’s prevention of the emergence of rifampicin resistance (Muller-Serieys et al., 2009).

##### 2.2.1.2 Fosfomycin

Fosfomycin remains the only phosphonic acid derivative that is used in the clinical setting (Silver, 2017), exhibiting broad-spectrum and bactericidal activity against gram-positive and gram-negative bacteria, including MRSA, and vancomycin-resistant enterococci (VRE) (Carlet and Mainardi, 2012). It exhibits hydrophilic properties and, in combination with its low molecular weight and diminished plasma protein binding, offers suitable distribution into tissue, including good bone penetration (Meißner et al., 1989; Silver, 2017; Morata and Soriano, 2019). According to several reports, fosfomycin, whether alone or in combination with other antibiotics, demonstrates the ability to penetrate and combat bacterial biofilms. This ability extends particularly to staphylococcal (Trautmann et al., 1992; Amorena et al., 1999; Monzón et al., 2003; Tang et al., 2012; Mihailescu et al., 2014), enterococcal (Alessandra et al., 2014) and extended spectrum beta-lactamase (ESBL) producing *E. coli* biofilms (Corvec et al., 2013; Wang et al., 2019), while also modifying the structure of their biofilm. While a good independent antibiofilm agent, with concentration – and dose-dependent activity, its effect is better shown at high doses (Tang et al., 2012). However, clinically high concentrations of the drug are probably toxic and not feasible, and the emergence of drug resistance further limits its standalone use. Therefore, combining fosfomycin with other antibiotics at recommended doses would be more practical and effective. Additionally, in conditions of high inoculum infections, such as some forms of osteomyelitis, fosfomycin’s activity may decrease (Mei et al., 2015), highlighting the necessity of combination therapy. Conveniently, reports suggest that fosfomycin can damage biofilm structure, thereby increasing the permeability of other antibiotics (Kusachi et al., 2011).

Activity against pseudomonal biofilms has also been shown in several studies, when combined with other antibiotics, including aminoglycosides and FQs (Kumon et al., 1995; Monden et al., 2002; Mikuniya et al., 2005, 2007; Cai et al., 2009; Anderson et al., 2013). In an *in vitro* study, fosfomycin exhibited successful inhibition levels only when used in combination with either gentamicin or ciprofloxacin. However, when administered alone, it failed to demonstrate significant inhibition (Wang et al., 2019).

It is recognized that rifampicin individually outperforms fosfomycin in inhibiting pseudomonal biofilm, but their combined administration demonstrates a higher efficacy in preventing biofilm formation (Liu et al., 2022). In fact, fosfomycin has been shown *in vitro* to enhance the activities of linezolid, minocycline, vancomycin and teicoplanin against MRSA as well (Tang et al., 2012). These combined treatments have proven be more effective than rifampicin combinations, offering potential therapeutic benefits and alternatives for infections related to catheters or prosthetic joints. However, it is worth noting that while fosfomycin may be used in combination with rifampicin against MRSA implant-associated infections, it cannot replace rifampicin as an antibiofilm agent (Mihailescu et al., 2014).

What makes the drug suitable for adjunctive treatment of implant associated infections is not only its ability to break up biofilms, allowing better absorption of other antibiotics (Kastoris et al., 2010), but also its presumed immunomodulatory effects (Raz, 2012) and occasional extended post-antibiotic effect (Mazzei et al., 2006). The frequency of adverse effects also appears to be low, primarily involving gastrointestinal disturbances and electrolyte imbalances, notably hypernatremia and hypokalaemia (Hashemian et al., 2019). Severe bone infections usually complicate with tissue hypoxia and formation of abscesses, protecting bacteria against some antibiotics. Fosfomycin’s increased antimicrobial effectiveness in environments with low oxygen levels and acidic pH, coupled with its ability to adequately penetrate abscess tissue, could offer advantages in managing these challenging-to-treat infections (Inouye et al., 1989; Sauermann et al., 2005; Hirakawa et al., 2018).

Although fosfomycin appears to possess the qualities necessary to be an effective component in antibiofilm therapy combinations, few clinical practice reviews or studies have been conducted in this regard, especially in the case of PJIs. A recent review of 365 patients with bone and joint infections concluded that when IV fosfomycin was used, either alone (6.3%) or in combination with another antibiotic (93.7%), 82.2% of cases were considered successfully treated (Tsegka et al., 2022), though most of these were cases of osteomyelitis, rather than PJIs. The reported toxicity level was low, and, in many cases, the IV treatment was completed by oral administration of antibiotics.

There are several ongoing prospective studies researching IV fosfomycin for the treatment of patients with osteoarticular infections (Karbysheva et al., 2019; Bodmann et al., 2021). Based on available data, IV fosfomycin may be an attractive choice for empiric and targeted first-line therapy (Vossen et al., 2020), but also presents as a viable therapeutic option in cases that fail to respond to initial treatment or when challenging-to-treat pathogens present.

##### 2.2.1.3 Tetracyclines

The tetracyclines are a broad-spectrum, bacteriostatic class of antibiotics that function as protein synthesis inhibitors, targeting the 30S ribosome of bacteria, preventing binding of tRNA (Grossman, 2016). They are considered effective against both gram-positive and gram-negative bacteria, but within biofilm-associated infections, most evidence surrounds their use as alternative therapy for gram-positive microorganisms. Data regarding bone penetration seems to be insufficient and conflicting. Older reports suggested that drugs such as tigecycline exhibited poor bone penetration (Moenster et al., 2013), whereas the long-acting derivatives of tetracycline, doxycycline, and minocycline, have excellent tissue penetration (Klein and Cunha, 1995). Tetracyclines exhibit high binding affinity to calcium, hence a high affinity for bone mineral matrix. The impact of tetracycline, minocycline, and doxycycline on bone cell signaling and their effects on the natural progression and balance of skeletal maturation remain uncertain (Warner et al., 2022). It is known however that retention in the bone is high, even after termination of treatment. In general, it is also recognized that tetracyclines exhibit higher tissue and cell penetration than some other classes, such as the beta-lactams or aminoglycosides (Moenster et al., 2013; Wu et al., 2014).

Evidence of anti-biofilm activity within these drugs exists, albeit limited and confined to gram-positive microorganisms. *In vitro* studies with minocycline and tigecycline showed superior anti-biofilm activity against *S. aureus* when compared with vancomycin and even linezolid (Issam et al., 2007). This effect was significantly enhanced when paired with rifampicin. Anti-biofilm activity against *Staphylococcus* spp., including MRSA and *S. epidermidis*, has been consistently shown in several analyses, particularly as part of a combination regimen (Rose and Poppens, 2009; Wu et al., 2014; Rosman et al., 2021; Doub et al., 2022). This is also true for vancomycin-sensitive enterococci (VSE) and VRE, for which tigecycline has shown to be particularly useful. One study evaluated multiple antibiotics commonly used in PJIs on a biofilm model of *S. aureus* derived from clinical strains and found that both rifampicin and tigecycline exhibited the highest eradication activity. However, as expected, achieving this required significantly higher MBEC compared to the MIC data (Molina-Manso et al., 2013).

There are a few concerns on the drug interaction between doxycycline with rifampicin since it may decrease the serum concentration of doxycycline (Colmenero et al., 1994). This effect is not observed with minocycline, and even so, numerous cases, clinical trials, and expert recommendations suggest that the combination of doxycycline and rifampicin is an effective regimen for various infectious diseases. *In vitro* data also suggest that minocycline has a better anti-staphylococcal activity than doxycycline (Klein and Cunha, 1995), but clinical superiority has not been demonstrated. Also, some gram-positive pathogens seem to be resistant to doxycycline but not minocycline, as the resistance mechanisms are not associated with resistance to the whole class (Thompson and Townsend, 2011; Doub et al., 2022).

Clinical use of tetracyclines has focused mainly on suppression therapy following surgical debridement and implant retention. A few cohorts have demonstrated adequate suppression rates of infection with these agents (Pradier et al., 2018; Leijtens et al., 2019; Sandiford et al., 2020; Ceccarelli et al., 2023). Also, it should be noted that a substantial number of patients in these studies that experienced infection relapse had to suspend the suppressive antibiotic weeks or months beforehand due to adverse effects. An American study achieved a 1-year event free rate of 88% with patients on oral doxycycline or minocycline after surgical debridement and implant retention (Jang et al., 2024). The simplicity and effectiveness of minocycline or doxycycline based chronic oral antimicrobial suppression, along with their favourable bioavailability and economic sustainability, make these drugs attractive options in patients with susceptible pathogens who are ineligible for standard surgical treatment of PJIs, particularly in staphylococcal infections. Additionally, some authors adopted the 100 mg q.d. dosage, instead of the recommended b.i.d., as to reduce potential side effects, and achieved favourable results (Leijtens et al., 2019).

Tetracyclines are also indicated as alternative drugs in the acute treatment of staphylococcal PJIs, as part of a combination regimen with other agents, such as rifampicin or FQs (Osmon et al., 2013). In this regard they are usually used as secondary companion drugs when *in vitro* susceptibility, allergies, intolerances, or potential intolerances thwarts the use of a quinolone. Minocycline-rifampicin can be used as treatment for MRSA, as well as tigecycline-rifampicin or even minocycline-fosfomycin (Perez-Jorge et al., 2016). Tigecycline is the only agent active against MRSA with simultaneous activity against gram-negative *Enterobacterales*. However, it lacks activity against *P. aeruginosa* (Rice, 2006). It could therefore have an important use in polymicrobial infections, which are a common challenge in the diabetic or immunocompromised patient. Minocycline has also shown favourable outcome against MRSA PJIs when combined with vancomycin (Bart et al., 2020).

The high affinity of tetracyclines for the bone mineral matrix, which are retained at elevated levels even after treatment interruption, could represent an element favouring the anti-biofilm activity tetracyclines in this setting. On the other hand, they exhibit bacteriostatic properties, which could be a disadvantage, and should not be administered to pregnant women or small children (Cross et al., 2016), which represent a minority in the PJIs setting. Conversely, while these drugs are less studied for treatment of staphylococcal PJIs, they offer ease of administration through a well absorbed oral route and a favourable side effect profile.

#### 2.2.2 Antibiotics active against gram-positive microorganisms

##### 2.2.2.1 Fusidic acid

Fusidic acid (FA) disrupts bacterial protein synthesis through binding of elongation factor G, a GTPase coded by the *fusA gene,* which is chromosomally located. It has a narrow spectrum of action, being active mainly against gram-positive microorganisms, namely Staphylococci, including MRSA, Vancomycin Intermediate *Staphylococcus aureus* (VISA) and heterogeneous VISA, and Streptococci. Classically, it has been considered inactive against Enterococci, but recent *in vivo* evidence suggests it may be an option to consider (Abdelmassih et al., 2024).

FA is available in topical, oral, and IV formulations, has good tissue penetration and high bioavailability when taken orally. It is a safe and well-tolerated drug. The most common adverse effects relate to the gastrointestinal tract and serious adverse effects are rare, most often cholestasis and citopenias (Christiansen, 1999). Concerns about drug–drug interactions exist but documentation is scarce. Nevertheless, coadministration with statins should be approached cautiously due to a significant risk of rhabdomyolysis (Gupta et al., 2016; Rönnqvist et al., 2018).

Its narrow spectrum of action, high bioavailability and adequate tissue penetration make it an attractive option for PJIs treatment, particularly in the setting of emerging antimicrobial resistance. However, monotherapy with fusidic acid has been associated with spontaneous chromosomal mutations and selection of resistant Staphylococci and Streptococci clones, limiting its usefulness as a single agent. This problem has been amplified by widespread use of topical formulations for skin and ophthalmic infections. Some authors have argued for the elimination of topical FA formulations from markets to preserve this option for systemic treatment of drug resistant pathogens, which may be a reasonable approach (Howden and Grayson, 2006). A recent meta-analysis has demonstrated increasing FA resistance among *S. aureus* isolates worldwide, particularly for MRSA and the American and Asian continents. Europe and Oceania had the lowest rates of FA resistance, but there may be some bias associated with MIC breakpoint heterogeneity between different continents (Hajikhani et al., 2021).

Predictably, resistance to FA is higher in biofilm isolates when compared to bacteria in the planktonic state (Saginur et al., 2006). *In vitro* evidence is heterogeneous, and some argues against the effect of FA in monotherapy in preventing biofilm maturation (Marquès et al., 2015).

Early recommendations for treatment of PJIs advocated for FA and rifampicin combination therapy (Zimmerli et al., 2004). Combination therapy may reduce the development of resistance by reducing the frequency of spontaneous resistance mutations. *In vitro* evidence suggests reduced likelihood of resistance development with concentrations of fusidic acid equivalent to doses of 500 mg t.i.d and that the combination with rifampicin may decrease the risk of resistance to the latter in Methicilin-Susceptible *Staphylococcus aureus* (MSSA), MRSA, VISA, and hetero-VISA populations (O’Neill et al., 2001; Hung-Jen et al., 2013).

To date, clinical evidence is mostly retrospective, with small numbers of patients and variable results. It clearly favours combination therapy (Drancourt et al., 1997; Ferry et al., 2009). Still, treatment failure with rifampicin and FA combination therapy in the literature is highly variable, though results are more favourable when fusidic acid is administered thrice daily (Drancourt et al., 1997; Aboltins et al., 2007; Ferry et al., 2009; Peel et al., 2013).

Lastly, a recent RCT identified a rifampicin-FA drug interaction, leading to decreased levels of FA. The levels of the latter decreased with continuing exposure, leaving the patients in effective rifampicin monotherapy, with increased risk of antibiotic resistance (Pushkin et al., 2016).

*In vitro* evidence for alternative combinations includes fosfomycin, tetracyclines, linezolid and daptomycin. Combination with fosfomycin has demonstrated significant synergism among *S. aureus* strains (Yu et al., 2010; Tang et al., 2012). Tetracyclines may also be an option, considering *in vitro* findings of minocycline and FA synergism (Wu et al., 2012). Both static and dynamic *in vitro* assessments support the association of FA with linezolid or daptomycin (Wafi et al., 2018). Linezolid and fusidic acid may even be applicable to *Enterococcal* infections (Ermertcan et al., 2010). However, clinical validation is required and there may be a legitimate concern for enhanced haematological toxicity when combining these two agents.

In sum, FA may be an interesting option for gram-positive PJIs, but its use should consider emerging resistance patterns and drug–drug interactions. Its association with rifampicin is poorly established and clinical evidence is scarce. Evaluating alternative combinations may be the path to rescuing this drug from present setbacks.

##### 2.2.2.2 Glycopeptides (vancomycin and teicoplanin)

Glycopeptides inhibit the synthesis of peptidoglycan, thus disrupting the cell wall formation of gram-positive microorganisms. This group includes vancomycin, which can be administered orally and intravenously, and teicoplanin, which can be administered both by IV infusion and intramuscular injection.

Vancomycin has long been regarded as a staple option for the treatment of PJIs in the setting of antibiotic resistance, particularly in MRSA and enterococcal infections (Osmon et al., 2013). Due to lack of absorption in the gastrointestinal tract, it must be infused for the treatment of PJIs. It is irritating to tissues and may cause thrombophlebitis. Infusions should be slowly paced to avoid anaphylactoid reaction and therapeutic drug monitoring (TDM) is recommended, due to considerable nephrotoxicity and ototoxicity, as is the avoidance of concurrent administration of nephrotoxic or ototoxic drugs. Ideal trough levels for the treatment of PJIs have been extrapolated from other complicated infections (15–20 mg/L), as there are no RCTs to guide clinical practice so far.

*In vitro* evidence on the combination with rifampicin for biofilm elimination is conflicting and there is some concern regarding the risk of rifampicin resistance development with this combination with supporting clinical evidence, though the latter is not specific to PJIs (Saginur et al., 2006; Hung-Jen et al., 2013; Tremblay et al., 2013). Alternatively, the combination of vancomycin or other peptide antibiotics with fosfomycin may be worthy of attention, considering favourable *in vitro* and *in vivo* evidence (Tang et al., 2011, 2012; Shi et al., 2014; Yu et al., 2020). However, vancomycin’s safety issues and complex PK/PD profile, requiring regular TDM and close surveillance of side effects make this option less than ideal, particularly for maintenance therapy.

Teicoplanin could be a reasonable alternative due to the possibility of transitioning patients to outpatient parenteral antibiotic therapy (OPAT). Both administration and TDM are less frequent, and it is slightly less nephrotoxic. However, available evidence and clinical experience is scarce (López Sánchez et al., 2016) and adverse effects are relevant and frequent, even with modified routes of administration, particularly in patients with previous adverse reactions to vancomycin (Peeters et al., 2016; Kim et al., 2019).

The newer lipopeptides and lipoglycopeptides are attractive alternatives to classic glycopeptides due to their long half-life, diminished nephrotoxicity, and infrequent side effects. Despite requiring IV administration, dosing intervals enable transition to OPAT. Existing evidence, though scarce, supports their bactericidal and antibiofilm effects against gram-positive cocci, such as Staphylococci, including MRSA, VISA and Vancomycin Resistant *Staphylococcus aureus* (VRSA), Streptococci and Enterococci, including VRE, as well as their applicability to the treatment of PJIs.

##### 2.2.2.3 Lipopeptides (daptomycin)

Among the newer lipopeptides and lipoglycopeptides, most research has been produced on daptomycin. Unlike other antibiotics that target the cell wall, which require active cell division to be effective (such as beta-lactams), daptomycin causes rapid depolarization of membrane potential and seems to be active even against microorganisms in a stationary-phase, rendering it an interesting option for infections where biofilm is relevant (Mascio et al., 2007). It is administered intravenously and has a short half-life. Tissue penetration is good, but it is important to recall that due to its inactivation by lung surfactant, daptomycin is not an option when treating patients with concomitant pneumonia.

Toxicity and severe side effects are less frequent when compared to glycopeptides. Eosinophilic pneumonitis, albeit rare and poorly understood, has been observed among patients treated with daptomycin. Myolysis is a more frequent side effect, particularly in the setting of renal dysfunction, prolonged treatment times or coadministration of statins (Ivor et al., 2012; Chang et al., 2017; Telles et al., 2019; Carli et al., 2020). In these cases, weekly creatine phosphokinase monitoring is recommended. Lastly, hepatotoxicity is rare but should be considered.

Daptomycin’s bactericidal effect has been shown to be superior to vancomycin in *S. aureus* models (Smith et al., 2009). The same is true for both its bactericidal and antibiofilm effects in *S. epidermidis in vitro* models. It may even have a similar or superior antibiofilm effect when compared to rifampicin, as shown in *in vitro S. epidermidis* biofilm models (Leite et al., 2011).

Clinical experience with daptomycin in PJIs started off on the wrong foot (Rao and Regalla, 2006) but the poor results of this initial study have since been attributed to insufficient dosing. More recent trials, including one RCT and other clinical trials of both retrospective and prospective design have established the efficacy and safety of doses between 6 and 9 mg/kg/day in the treatment of PJIs.

Clinical trials so far have assessed daptomycin monotherapy. However, combinations with rifampicin and clarithromycin seem to produce relevant bactericidal and antibiofilm effects in *S. aureus in vitro* models (Parra-Ruiz et al., 2010; Snyder et al., 2014). A combination with fosfomycin also seems to effectively target vancomycin-resistant Enterococci *in vitro* (Tilman et al., 2015)This combination needs clinical validation, but there is some evidence to its effectiveness in enterococcal and staphylococcal bacteraemia (Pujol et al., 2020; Tseng et al., 2023) as well as anecdotal evidence as to its use in PJIs (Luengo-Alonso et al., 2018).

##### 2.2.2.4 Lipoglycopeptides (oritavancin, dalbavancin, and telavancin)

Lipoglycopeptides inhibit bacterial cell wall synthesis and disrupt cell membrane integrity. They are available in IV formulations and oritavancin and dalbavancin have exceptionally long half-lives, making them extremely attractive for OPAT. Adequate tissue penetration and a favourable safety profile make them an interesting option for treating PJIs due to multidrug-resistant (MDR) gram-positive microorganisms. Although significantly less frequent, side effects are like those of vancomycin, including the possibility of anaphylactoid reaction and nephrotoxicity. Dalbavancin has also been associated with hepatotoxicity.

There is few evidence regarding oritavancin use in PJIs. However, biological plausibility favours this approach. *In vitro* evidence demonstrates bactericidal and antibiofilm activity in *S. aureus* models, including MRSA and VRSA (Yan et al., 2018) as well as possible synergism with rifampicin, gentamicin, and linezolid (Lin et al., 2014; Yan et al., 2018).

A recent review highlights the possible role of oritavancin in device-related infections, cataloguing clinical evidence with a total of 29 PJIs cases (Lupia et al., 2023). Notwithstanding, to date, there have been no RCTs and despite it being clear that sequential dosing is necessary, the ideal regimen in PJIs remains unclear, thus undermining the applicability of oritavancin in a clinical context.

Dalbavancin is another option to consider with *in vitro* activity against Staphylococci and Streptococci and synergism with rifampicin. Both seem to be superior to the effects observed with vancomycin (Di Pilato et al., 2019; Jacob et al., 2021). Off-label use of dalbavancin to treat PJIs is being increasingly adopted (Wunsch et al., 2019) and clinical experience with two doses of 1,500 mg seems promising (Simon et al., 2022; Dimopoulou et al., 2023; Doub et al., 2023). However, data regarding the safety and need for long term therapy with dalbavancin is lacking.

Telavancin is the least established lipoglycopeptide in the treatment of PJIs. While there is some evidence as to its bactericidal and antibiofilm activity in both *in vitro* and *in vivo* models (Chan et al., 2015) as well as possible synergism with rifampicin (Jahanbakhsh et al., 2018), clinical data of telavancin’s applicability in bone and joint infections is scarce and mostly retrospective (Harting et al., 2017; Reilly et al., 2020; Sims et al., 2021), which may be particularly relevant in the setting of PJIs due to long treatment times and patient characteristics.

#### 2.2.3 Antibiotics active against gram-negative microorganisms

##### 2.2.3.1 Colistin

The activity of polymyxin E (i.e., colistin) against gram-negative microorganisms’ biofilm has been shown in multiple *in vitro* and *in vivo* models. Most of these studies however reviewed colistin’s activity on *Pseudomonas aeruginosa* biofilms (Haagensen et al., 2007; Pamp et al., 2008; Cai et al., 2010; Herrmann et al., 2010; Wang et al., 2011; Chiang et al., 2012; Wang et al., 2012; Brochmann et al., 2013; Chambers and Sauer, 2013; Lora-Tamayo et al., 2014). Still, activity against some *Enterobacteriaceae* such as *E. coli* has also been demonstrated (Klinger-Strobel et al., 2017). Instead of targeting the metabolically active outer cell layer of the biofilm, as other antibiotics such as the fluroquinolones and aminoglycosides do, colistin seems to target the inner, less metabolically active layer of the structure (Klausen et al., 2003; Haagensen et al., 2007; Pamp et al., 2008).

As referred previously, virtually all antibiotics are less active against biofilm-coated bacteria than against their planktonic counterpart. To an extent, this is also true for polymyxins against gram-negative bacilli. One *in vitro* model revealed a MBIC concentration 4–8 times higher than the usual MIC of 2 mg/L (Wang et al., 2011), which was also confirmed in an *in vivo* mice model. However, achieving the necessary plasma colistin concentrations through IV administration of colistimethate sodium (CMS), the prodrug of colistin, is deemed unlikely (Nation and Li, 2009; Plachouras et al., 2009; Garonzik et al., 2011). The concerns that already exist in achieving sufficiently high colistin concentration for planktonic infections can therefore be extended to biofilm-associated infections. Sub-inhibitory concentrations are of course related to therapeutic failure and resistance selection (Klausen et al., 2003; Plachouras et al., 2009; Lora-Tamayo et al., 2014). In consideration of these PK/PD problems, current recommendations suggest the association of high dose CMS with a second antimicrobial (Nation and Li, 2009; Bergen et al., 2011), on the basis that each drug could work together to target distinct parts of the bacterial population within the biofilm architecture. Also, it is suggested that colistin’s mode of action synergizes with other antibiotics, as it acts on the bacterial external membrane, enhancing its own uptake along with other compounds (Hancock and Wong, 1984; Hancock, 1997; Bergen et al., 2011). Combined treatments intend to override resistance to colistin or other antimicrobials, while maintaining adequate doses and reducing toxicity associated with colistin. Previous studies have shown this synergy against gram-negative pathogens, with an associated reduced hospital mortality (Batirel et al., 2014).

Multiple studies have shown a combined effect of colistin-carbapenems on biofilm structures of gram-negative bacteria, such as *P. aeruginosa* or *A. baumannii*, particularly colistin-meropenem dual therapy (Copur et al., 2022; Taşkın Kafa and Hasbek, 2022) but also colistin-doripenem (Lora-Tamayo et al., 2014).

Limited clinical experience exists for colistin’s use in treating biofilm-related infections, as it is usually regarded as last-line therapy. Colistin has been administered via various methods, including aerosolized and IV routes, as well as locally through intraventricular administration or in cement spacers for CNS or prosthetic joint infections. A lack of oral option for prolonged treatment is a downside of the drug. Moreover, optimal dosage and comparative efficacy between colistin alone or in combination have not been adequately assessed (Lora-Tamayo et al., 2019). Most current knowledge aspires from experience regarding cystic fibrosis patients, where colistin is used as first line treatment against *P. aeruginosa* infections, as well as salvage therapy for MDR strains (Döring et al., 2000; Heijerman et al., 2009). Most clinical experience refers to the use of nebulized colistin, with minimal information on IV usage (Conway et al., 1997; Ledson et al., 1998; Döring et al., 2000; Heijerman et al., 2009).

In the orthopaedic device infection setting, colistin has also been used as last-line treatment, but its true efficacy and potential benefits of combined therapy are yet to be rigorously evaluated. Previous studies indicated poor bone diffusion with this drug (Falagas and Kasiakou, 2005), leading to its specific administration in local beads and cement spacers (Rosenthal et al., 1976; Murray, 1984). However, clinical experience with IV CMS is scarce and mainly approaches its use in last-line therapy of complicated bone and joint infections caused by MDR gram-negative microorganisms. A retrospective cohort of nineteen patients evaluated the use of colistin for challenging osteoarticular infections caused by MDR/ extensively drug resistant (XDR) pathogens. Twelve of these, were implant-associated. IV colistin was used alone as salvage therapy in 90% of cases. A reported 74% of patients experienced clinical remission, however the implant-associated group experienced a 42% rate of treatment failure (Valour et al., 2012). Another review of twenty-two cases of osteoarticular infections caused by XDR *P. aeruginosa*, of which fifteen involved orthopaedic devices, concluded that 80% of patients achieved a cure rate when colistin was associated with a β-lactam, compared to 29% in the monotherapy group. Interestingly, this combination was deemed superior when the β-lactam was administered continuously, rather than in bolus (Lora-Tamayo et al., 2019).

Concern around colistin exists considering the frequency of its main side effects. Reviews estimate that up to 60% of patients can experience some degree of nephrotoxicity, which usually manifest in the first 2 weeks of administration (Shahbazi and Dashti-Khavidaki, 2015). Neurotoxicity is also a known but rare side effect, with an estimate of 6% of patients experiencing paraesthesia, vertigo, visual disturbances, hallucinations, mental confusion, or seizures. It is important to verify patient risk factors for these adverse events and keep close monitoring as to avoid serious repercussions.

Colistin shows promise in treating orthopaedic device-related infections caused by MDR or XDR gram-negative bacteria, particularly when used alongside other antimicrobials. When dealing with *P. aeruginosa* exhibiting resistance or intermediate susceptibility to β-lactams, combining colistin with a β-lactam can enhance infection outcomes. Nonetheless, further research is necessary to validate these findings and investigate alternative treatment combinations.

## 3 Discussion

The biofilm principle has modified the way we view and treat device-related infections. Modern treatment of these infections calls for a diverse arsenal of antibiotic compounds that, not only provide unequivocal antibiofilm properties, but take into account multiple factors that can tailor to patients’ individual needs. As previously stated, rifampicin and FQs have been the most investigated drugs in this setting, particularly for PJIs. However, as demonstrated beforehand, there are numerous other classes and agents that should be considered for treatment and included in major comparative studies.

Considering the rapid formation of biofilm in PJIs, the initial choice of antibiotic regimen must provide unequivocal antibiofilm ability to penetrate the biofilm matrix and eradicate the bacteria embedded in it. Adequate penetration into this structure is crucial to ensure the antimicrobial’s effectiveness and potentially enhance the effects of other antimicrobials, providing synergistic characteristics.

Treatment for PJIs requires optimal antibiotic coverage, particularly during the initial postoperative weeks. Despite the reduction in bacterial load post-surgery, administering high-dose antibiotics and optimizing PK/PD parameters yields improved infection control (Guilhaumou et al., 2019) and decreases the likelihood of bacterial resistance emergence. To mitigate the selection of resistant strains and consequent treatment failure, the initial treatment regimen should incorporate bacterial load-independent drugs with a minimal risk of resistance development – such as β-lactams, vancomycin, or daptomycin (Vavasseur and Zeller, 2022). IV administration facilitates the use of higher doses, circumvents the intestinal and hepatic first-pass effect that hinders the bioavailability of certain drugs (Zeller et al., 2021), and avoids malabsorption issues related to gastrointestinal intolerance and some interactions with medications. How long the initial IV regimen should be administered remains controversial, with recent evidence pointing to similar efficacy for shorter IV antibiotic courses (Ho Kwong et al., 2019).

Eradicating biofilm requires higher and continued antibiotic doses, and even this often does not successfully eradicate biofilm infections (Liu et al., 2022). Conventional antibiotic therapy against these infections is particularly challenging since only doses that are sublethal to the biofilm can be delivered safely to patient. Current literature strongly suggests that elimination of biofilms is better accomplished by using combinations of antibiotics, rather than single therapy (Valerius et al., 1991; Döring et al., 2004; Høiby et al., 2005; Pamp et al., 2008; Gbejuade et al., 2015; Taha et al., 2018). Besides increasing efficacy and providing good coverage for empiric therapy, this strategy reduces drug doses, toxicity issues and minimizes emergence of resistances (Neut et al., 2006; Hagihara et al., 2012; Worthington and Melander, 2013; Taha et al., 2018).

Current research on biofilm formation and elimination is scarce, technically complex and hampered by a heterogenous use of techniques and definitions. The behaviour of biofilm and the natural history of infection vary greatly among different pathogens, further complicating experimental models. Most studies that assess antibiofilm activity rely on *in vitro* models, which can be divided into three categories: static, dynamic and microcosm. Static models are conventional plate-based assessments, where metabolites and other products accumulate rather than flow. These are cost-effective and allow for multiple simultaneous tests but fall short of mimicking real-life conditions. Dynamic models are closer to biological conditions since they allow for the addition of nutrients and clearance of waste products. Microcosms build on the latter, adding environmental modelling to approximate *in situ* conditions (Roy et al., 2018). Nonetheless, conventional microbiological assessments do not necessarily correlate with clinical outcomes and clinical validation of *in vitro* models or animal studies is still a work in progress. Accordingly, the complexity and heterogeneity of biofilm assessment techniques has precluded its inclusion in routine laboratory testing.

Given the rise of antimicrobial resistances, long treatment times and subsequent tolerance issues, as well as the particularities of those with PJIs, namely increasing age, frequent polypharmacy, with the added difficulty of drug–drug interactions, the available antimicrobial arsenal should be as diverse as possible to facilitate a tailored and practical intervention. As the prevalence of MDR microorganisms continues to escalate, along with the noted rise in resistance levels to traditional antibiofilm drugs, like rifampicin and FQs (Benito et al., 2016; Eriksson et al., 2023), there is a growing demand for alternative therapies. However, due to the predominance of *in vitro* studies and the absence of large-scale comparative research, formulating practical clinical recommendations for alternative drugs has proven particularly challenging.

Another essential consideration is ensuring adequate tissue penetration and, in the case of PJIs treatment, adequate bone absorption. Additionally, longer half-lives offer the advantage of reduced dosing frequency. Access to oral formulations with good bioavailability is also a key factor, since many of these infections demand prolonged treatments, which can lead to unnecessary extended hospitalizations. Solid oral options can hence reduce the risk of inpatient complications and provide a more convenient approach.

Our preferred treatment of choice for gram-positive PJIs includes a short course of IV beta-lactams followed by oral step-down therapy combining an antibiofilm agent, preferably rifampicin. In the case of gram-negative PJIs we prefer the FQs, particularly ciprofloxacin. Whenever possible, we favour the combination of rifampicin and fluoroquinolones for the definitive treatment of gram-positive PJIs.

Nonetheless, possible combinations are multiple and the search for synergy within antibiotics is important. A summary of the antibiofilm activity of reviewed antibiotics can be found in Table 1. Conventional step-down options include quinolones, cotrimoxazole, doxycycline, clindamycin, minocycline and oxazolidinones, the latter being the least preferred due to relevant myelotoxicity and thus reserved for MDR infections where other options are not viable. Despite being a first-line option in the early phase of PJIs, step-down with beta-lactams has often been regarded with suspicion due to their time-dependent bactericidal activity and low bioavailability when administered *per os*. Long term courses of IV antibiotics are reserved for when resistance patterns preclude other options.

**Table 1:** Summary of antibiofilm activity of reviewed antibiotics.

	Rifampicin	Fluoroquinolones	Fosfomycin	Tetracyclines	Fusidic acid	Glycopeptides - Vancomycin - Teicoplanin	Daptomycin	Lipoglycopeptides - Oritavancin - Telavancin - Dalbavancin	Colistin
**Antibiofilm activity**	Gram + Staphylococci	Gram + Gram − Enterobacter and Pseudomonas. For Staphylococci prefer levofloxacin or moxifloxacin	Gram + Gram − Mainly Gram + (including MRSA) and Enterococci (including VRE when combined with daptomycin) Anti-pseudomonal biofilms in association with ciprofloxacin or gentamicin.	Gram + Gram − Mainly Gram + Doxycycline and minocycline for Staphylococci	Gram + Specially Staphylococci	Gram + Staphylococci (MRSA) and Enterococci	Gram + Staphylococci (including MRSA) and Enterococci (including VRE when combined with fosfomycin)	Gram + Oritavancin and dalbavancin against Staphylococci (including MRSA) Less evidence for the use of telavancin	Gram −
**Administration**	*Per os* and IV	*Per os* and IV	*Per os* bioavailability not suitable for PJI infection	*Per os* and IV	*Per os* and IV	*Per os* bioavailability not suitable for PJI infection	IV only – for PJI use 6–9 mg/kg/day	IV only	IV only
**Caveats**	Never use in monotherapy. When possible, associate with fluoroquinolones. Preferably not associated with Linezolid or cotrimoxazole. Should not be used empirically. Start a few days after IV-ATB and only after surgical drains removed and no drainage from the wound.	Preferably do not use in monotherapy. Against Gram+, when possible, use with rifampicin. Start a few days after IV ATB and only after surgical drains removed and no drainage from the wound.	Use always in combination, especially with linezolid, minocycline, vancomycin and teicoplanin against MRSA.	Preferred antibiotics for suppressive therapy.	Caution when associate with rifampicin. Use always in 500 mg TID dose. Combinations includes fosfomycin, tetracyclines, linezolid and daptomycin.	Avoid association with rifampicin.			Only in salvage therapy in *Pseudomonas aeruginosa* and *Acinetobacter baumannii* in combination with meropenem.

There is still insufficient evidence as to how long the initial IV regimen should be administered. The trend is to shorten IV therapy as much as possible to avoid catheter-associated complications, shorten inpatient stay, lower costs, and enhance patient comfort. The results of the OVIVA trial have provided insight on the noninferiority of oral therapy when used in the first 6 weeks for complex orthopaedic infection, as assessed by 1-year treatment failure (Ho Kwong et al., 2019). IV antibiotics can likely be transitioned to an oral regimen early on, provided an antibiotic with effective antibiofilm properties and sufficient bioavailability is used.

Regarding total duration of antibiotic therapy, little consensus exists, for it appears to be as variable as 2 – 12 weeks, depending on severity, location, procedure, or comorbidities. Bernard et al. recently described that 6 weeks was not non-inferior to 12 weeks on patients with microbiologically confirmed prosthetic joint infections that were managed with standard and appropriate surgical procedures (Bernard et al., 2021). Numerous questions remain regarding the optimal duration for PJIs; it must be tailored to the surgical approach (Vavasseur and Zeller, 2022). The results from the SOLARIO trial might shed some light on the matter (Dudareva et al., 2019).

In conclusion, biofilm-related infections pose a diagnostic and treatment challenge. An increase in the number of these infections is anticipated, paralleling the projected rise in prostheses implantation due to a continuously aging population. Antimicrobial tolerance and the constant emergence of adaptive resistance mechanisms in bacteria embedded in this complex matrix calls for a wide selection of effective biofilm-active antibiotics. Applying biofilm models for susceptibility testing in routine laboratory settings lacks clinical utility. However, in the future, it would be ideal if antibiotic selection could be tailored based on the specific characteristics of the biofilm, including the bacterial strain involved and the way the biofilm integrates into the host. While rifampicin and fluoroquinolones have historically been key treatments for biofilms, other effective drugs exist, highlighting the need for large-scale comparative trials. A comprehensive and flexible approach, incorporating both established and emerging therapies, is fundamental for effective management and improved patient outcomes.
